# Postbiotic Effects of *Pediococcus acidophilus* LS for Anti-Melanogenesis, Photoprotection, and Wound Repair

**DOI:** 10.3390/microorganisms13092207

**Published:** 2025-09-20

**Authors:** Chiung-Hung Chang, Jai-Sing Yang, Yen-Ju Lai, Bi Yu, Yuan-Man Hsu

**Affiliations:** 1 Department of Traditional Chinese Medicine, Tainan Municipal Hospital (Managed by Show Chwan Medical Care Corporation), Tainan 701033, Taiwan; changch99@gmail.com; 2 Department of Acupressure Technology, Chung Hwa University of Medical Technology, Tainan 717302, Taiwan; 3 School of Chinese Medicine for Post-Baccalaureate, I-Shou University, Kaohsiung 824005, Taiwan; 4 Department of Medical Research, China Medical University Hospital, China Medical University, Taichung 404327, Taiwan; jaisingyang@gmail.com; 5 Department of Animal Science and Biotechnology, Tunghai University, Taichung 407224, Taiwan; u9978012@gmail.com; 6 Department of Animal Science, National Chung Hsing University, Taichung 402202, Taiwan; byu@dragon.nchu.edu.tw; 7 Department of Biological Science and Technology, China Medical University, Taichung 406040, Taiwan

**Keywords:** *Pediococcus acidophilus* LS, cell-free supernatant, melanogenesis, photodamage, wound healing

## Abstract

Skin health is significantly impacted by factors such as melanin production, UV-induced photodamage, and wound healing. Excessive melanin leads to hyperpigmentation, while UVA radiation accelerates skin aging and oxidative stress. This study investigated the multi-functional dermatological potential of *S* strain LS-derived cell-free supernatant (CFS-LS) to address these concerns. Our findings demonstrate that CFS-LS effectively inhibits melanogenesis in B16F10 cells. It significantly reduced α-MSH-induced melanin synthesis, comparable to arbutin, by downregulating key melanogenic enzymes (tyrosinase, TRP-1, and TRP-2) and regulatory proteins (p-CREB, MITF, SOX9, and SOX10). Mechanistically, CFS-LS suppressed the phosphorylation of MEK, ERK, p38, and JNK, indicating a dual inhibitory effect on both PKA/CREB and MAPK pathways. Furthermore, CFS-LS mitigated UVA-induced photodamage in HaCaT cells by significantly reducing intracellular reactive oxygen species and suppressing the downstream phosphorylation of p53 and α-MSH levels. It also restored UVA-suppressed Nrf-2 and HO-1 expression, enhancing cellular antioxidant defenses. Lastly, CFS-LS promoted skin wound healing by significantly enhancing HaCaT cell migration in a scratch assay, associated with increased p-MEK1/2 and p-ERK1/2 levels, and notably elevated collagen type I synthesis. Collectively, these results highlight CFS-LS as a potent multi-functional agent for skin protection and repair, with significant potential for cosmetic and therapeutic applications. The active components of CFS-LS warrant further investigation.

## 1. Introduction

Skin, hair, and eye color in mammals are mainly determined by melanin produced in the basal epidermis by melanocytes, which transfer the melanin to keratinocytes, forming the epidermal melanin unit [1,2]. Melanin protects the skin by absorbing ultraviolet (UV) radiation, preventing UV-induced damage [3]. However, excessive melanin causes skin aging, such as freckles and age spots, and increases melanoma risk [4]. UVA radiation, which constitutes 90–99% of UV reaching the Earth [5], penetrates deep into the dermis, damaging collagen and elastic fibers, stimulating melanin production, and accelerating skin aging [6]. UVA induces reactive oxygen species (ROS), causing oxidative DNA damage [7,8].

UV exposure elevates p53 in keratinocytes, promoting expression of pro-opiomelanocortin (POMC), which is cleaved into α-melanocyte-stimulating hormone (α-MSH). α-MSH binds melanocortin 1 receptor (MC1R) on melanocytes, increasing cAMP and activating protein kinase A (PKA). This leads to the upregulation of CREB and microphthalmia-associated transcription factor (MITF), which drives the expression of melanogenic enzymes such as tyrosinase and tyrosinase-related proteins (TRP-1 and TRP-2), resulting in melanin synthesis [3,9].

Repeated exposure to UV radiation is a primary cause of premature skin aging and increased risk of malignant skin cancers [10]. To combat these issues, a major focus has been placed on reducing hyperpigmentation and oxidative damage. Traditional tyrosinase inhibitors, such as hydroquinone, kojic acid, and arbutin, are widely used for this purpose. However, their application is limited by concerns regarding carcinogenicity, instability, and a lack of clear mechanistic understanding [11,12]. Consequently, there is an urgent need for novel, safe, and effective compounds with both anti-aging and anti-pigmentation properties.

In recent years, probiotic bacterial cell-free supernatants—filtered fluids containing bacterial metabolites but no live cells—have emerged as promising postbiotics that benefit skin health by reducing aging and pigmentation. These bioactive effects are mainly due to a complex mix of metabolites like organic acids, peptides, and phenolic derivatives produced by lactic acid bacteria [13]. For instance, studies have shown that supernatants from strains like *Lactobacillus acidophilus* and *Pediococcus acidilactici* exhibit anti-melanogenic and antioxidant activities [14,15,16,17,18]. However, the specific active compounds responsible for these effects remain unidentified. Thus, while the benefits are evident, the precise active ingredients remain largely unidentified.

Considering the established roles of postbiotics in skin protection, the potential of *Pediococcus acidophilus* strain LS-derived cell-free supernatant (CFS-LS) to reduce UV-induced photodamage, downregulate melanogenesis, and facilitate wound healing was evaluated. This study examined the effects of CFS-LS on melanogenesis-related signaling in B16F10 cells, antioxidant responses in UVA-irradiated HaCaT cells, and re-epithelialization processes in a scratch wound model. The results provide insight into the potential use of CFS-LS as a multi-functional agent for skin protection and repair.

## 2. Materials and Methods

### 2.1. Bacterial Strain and Culture Conditions

The *Pediococcus acidophilus* strain LS (LS) was isolated from the gastrointestinal tract of commercially sourced broiler chickens [19]. All procedures related to the use of animal samples were reviewed and approved by the Institutional Animal Care and Use Committee (IACUC) of National Chung Hsing University, Taiwan, under ethical approval number (IACUC No. 107-098). For growth analysis, a single colony was cultured overnight at 37 °C in de Man–Rogosa–Sharpe (MRS) broth (BD Biosciences, Ann Arbor, MI, USA) or modified MRS (mMRS) broth (Liofilchem, Waltham, MA, USA), with the latter prepared without Tween 80 from standard MRS. An overnight culture adjusted to OD_600_ = 0.1 was used to inoculate the fresh broths, and optical density was then measured every 3 h over a 24 h period. Results are presented as the mean of triplicate measurements. For functional analysis, LS was cultured in mMRS broth for 18 h, followed by centrifugation at 6000× *g* for 10 min. The resulting cell-free supernatant (CFS-LS) was collected, with the undiluted form designated as 100%.

### 2.2. Cell Culture

Mouse melanoma B16F10 cells (BCRC 60031) [20] were purchased from the Bioresource Collection and Research Center (BCRC), Taiwan, and human keratinocyte HaCaT cells (Elabscience CL-0090) [21] were obtained from Elabscience Biotechnology Inc. Both cell lines were cultured in Dulbecco’s Modified Eagle Medium (DMEM; Gibco-Life Technologies, Gaithersburg, MD, USA) supplemented with 10% cosmic calf serum (CCS; Hyclone, Logan, UT, USA), and penicillin (100 lU/mL)–streptomycin (100 μg/mL) solution (Corning Inc., Corning, NY, USA). Cells were maintained at 37 °C with 5% CO_2_.

### 2.3. Cell Viability Assay

B16F10 and HaCaT cells were seeded in 96-well plates at 1 × 10^4^ cells/well and incubated for 24 h at 37 °C with 5% CO_2_. Cells were then treated with 100 μL of diluted CFS-LS at the indicated concentrations and incubated for an additional 24 or 48 h. After treatment, 0.5 mg/mL MTT solution (Sigma-Aldrich, St. Louis, MO, USA) was added, and cells were incubated for 4 h. The medium was removed, and formazan crystals were dissolved in DMSO (Sigma-Aldrich, USA). Absorbance was measured at 570 nm. All experiments were performed in triplicate, and IC_50_ values were calculated from cell viability data using regression analysis.

### 2.4. Melanin Content Assay

B16F10 cells were seeded in six-well plates (2 × 10^5^ cells/well) and incubated at 37 °C with 5% CO_2_ for 24 h. Cells were then treated with DMEM containing 1 μM of α-MSH [22] (Sigma-Aldrich, USA), 300 μM of arbutin [23] (Sigma-Aldrich, USA), and various concentrations of CFS-LS for 48 h. After treatment, cells were lysed with 1 N NaOH containing 10% DMSO, heated at 95 °C for 1 h. The samples were allowed to cool at room temperature for 2–3 min, followed by centrifugation at 18,000× *g* for 1 min. The melanin content in the resulting supernatant was determined by measuring the absorbance at 405 nm with a Smart Microplate Reader (SMR16, USCN KIT, Inc., Wuhan, China). The results represent the means of triplicate samples, relative to untreated controls.

### 2.5. Fontana–Masson Staining Assays 

B16F10 cells were seeded in six-well plates at a density of 2 × 10^5^ cells per well and were treated as previously described in the melanin content assay. After treatment, cells were fixed with 10% formaldehyde in phosphate-buffered saline (PBS, pH 7.4) for 20 min at room temperature. Following fixation, cells were gently washed three times with PBS to remove residual fixative. The Fontana–Masson staining was performed using a Fontana–Masson Stain Kit (ScyTek Laboratories, Logan, UT, USA), according to the manufacturer’s protocol with modifications to ensure optimal staining. Briefly, 200 µL of freshly prepared ammoniacal silver solution, pre-warmed to 60 °C, was added to each well. The plates were incubated in a humidified chamber at 60 °C for approximately 30 min or until a yellowish-brown coloration developed, indicating silver impregnation. During incubation, care was taken to prevent the solution from drying out by maintaining sufficient humidity. Following silver impregnation, the cells were rinsed gently three times with distilled water, with each wash lasting 1 min. Subsequently, 200 µL of 0.2% gold chloride solution was applied for 30 s at room temperature to tone the silver deposits. The cells were again washed three times with distilled water. To fix the silver deposits and reduce background staining, 200 µL of 5% sodium thiosulfate solution was added for 1 min, followed by three washes with distilled water. A final wash was performed under running tap water for 2 min to thoroughly remove residual reagents. Cells were then counterstained with 200 µL of Nuclear Fast Red for 5 min at room temperature to visualize nuclei. After counterstaining, the slides were washed three times with distilled water. Dehydration was carried out rapidly through two changes of 95% ethanol (1 min each), followed by absolute ethanol for three changes of 1 min each. Finally, the stained samples were mounted with a synthetic resin mounting medium. Under microscopic examination, melanin and argentaffin granules appeared as black deposits, whereas nuclei were stained red. High-resolution images were captured using a Carl Zeiss digital microscope (Carl Zeiss, White Plains, NY, USA) under consistent illumination and magnification settings to ensure reproducibility and facilitate quantitative analysis [24].

### 2.6. Preparation of Cell Extracts and Western Blot Analysis

B16F10 cells were seeded into six-well plates at a density of 2 × 10^5^ cells per well. Protein samples were collected 24 and 48 h after treatment, as outlined in the melanin content assay protocol. For the HaCaT cell experiments, cells were seeded at 3 × 10^5^ cells per well and cultured for 24 h. They were subsequently pretreated with various concentrations of CFS-LS for an additional 24 h before being exposed to UVA irradiation at 5 J/cm^2^. After irradiation, the cells were maintained under standard culture conditions for 30 min before subsequent analyses. Cells were then washed with PBS, supplied with fresh serum-free medium, and collected. They were lysed with 10% SDS (Bio Basic, Markham, ON, Canada) and 5 μM of urea (Bio Basic, Canada). Protein concentration was measured using the BCA Protein Assay Kit (Thermo Fisher Scientific, Waltham, MA, USA). Equal amounts of protein (30–60 μg) were separated on an mPAGER 4–12% Bis-Tris Precast Gel (Merck KGaA, Darmstadt, Germany) and transferred to a PVDF membrane (Merck KGaA, Germany). The membrane was blocked with 5% non-fat milk in TBST (Tris-Buffered Saline with Tween 20) for 1 h at 37 °C, then incubated overnight at 4 °C with primary antibodies diluted 1:1000 in 10% BSA. Primary antibodies included β-actin (ARG62346, Arigo, Hsinchu, Taiwan), p38 (ARG55258, Arigo, Taiwan), p-p38 (ARG51850, Arigo, Taiwan), TRP-1 (ARG56122, Arigo, Taiwan), TRP-2 (ARG43800, Arigo, Taiwan), MEK (ARG65359, Arigo, Taiwan), JNK (ARG51218, Arigo, Taiwan), p-JNK (ARG51807, Arigo, Taiwan), ERK (ARG55797, Arigo, Taiwan), p-MEK (ARG52333, Arigo, Taiwan), p-ERK (ARG55797, Arigo, Taiwan), CREB (GTX112846, GeneTex, Irvine, CA, USA), p-CREB (GTX130379, GeneTex, Irvine, CA, USA), MITF (GTX113776, GeneTex, USA), tyrosinase (GTX16389, GeneTex, USA), HO-1 (GTX637432, GeneTex, USA), p53 (GTX638291, GeneTex, USA), SOX9 (sc-1665055, Santa Cruz, Santa Cruz, CA, USA), SOX10 (sc-365692, Santa Cruz, USA), Nrf-2 (sc-365949, Santa Cruz, USA), and p-p53 (sc-377567, Santa Cruz, USA). After overnight incubation, the membrane was washed twice with TBST and then incubated with HRP-conjugated secondary antibodies (1:5000; Jackson ImmunoResearch, West Grove, PA, USA) in 5% milk for 1 h at room temperature. After this incubation, the membrane was washed twice with TBST, and bands were detected using T-Pro LumiDura chemiluminescent substrate (T-Probio, Taiwan) and quantified via densitometry using the EvolutionCapt system (FUSION FX6, Vilber, Marne-la-Vallée, France).

### 2.7. UVA-Induced ROS Measurement in HaCaT Cells

HaCaT cells were seeded in black-walled 96-well plates (1 × 10^4^ cells per well) (Corning Costar, Thermo Scientific, Waltham, MA, USA) and incubated overnight at 37 °C under 5% CO_2_ to allow cell attachment and stabilization. After overnight incubation, cells were treated with various concentrations of CFS-LS for 24 h. Following treatment, cells were washed twice with phosphate-buffered saline (PBS, pH 7.4) to remove residual compounds. The culture medium was then replaced with phenol red-free DMEM supplemented with 10% charcoal-stripped serum (CCS). Cells were subjected to UVA irradiation at an energy dose of 5 J/cm^2^ (wavelength 365 nm) using a CL-1000 UV Crosslinker (UVP, Syracuse, NY, USA). The exposure duration was approximately 40 min. Cells were washed twice with PBS to remove residual CCS. After UVA exposure, they were incubated with 10 μM of CM-H_2_DCFDA (5-(and-6)-chloromethyl-2′,7′-dichlorodihydrofluorescein diacetate, acetyl ester; Abcam, Cambridge, UK) diluted in phenol red-free and serum-free DMEM, then incubated at 37 °C in the dark for 30 min. Cells were then washed twice with PBS to remove excess CM-H_2_DCFDA. Subsequently, fluorescence intensity, indicative of intracellular reactive oxygen species (ROS) levels, was measured using BioTek Cytation 5 Cell Imaging Multimode Reader (BioTek Cytation 5, Winooski, VT, USA), with excitation and emission wavelengths set at 485 nm and 528 nm, respectively. All measurements were conducted in triplicate, and data were expressed as the mean fluorescence intensity relative to the UVA-exposed untreated control group [25].

### 2.8. Measurement of α-MSH Expression

HaCaT cells were seeded in 6-well plates (3 × 10^5^ cells/well) and incubated for 24 h. Cells were treated with various concentrations of CFS-LS for another 24 h. After washing with PBS, the medium was replaced with phenol red-free DMEM containing 10% CCS. Cells were then exposed to UVA irradiation at 5 J/cm^2^. After irradiation, cells were returned to the incubator for 30 min. Culture supernatants were collected, and α-MSH levels were quantified using the Human α-MSH ELISA Kit (FineTest, Wuhan, China) according to the manufacturer’s instructions. Procedure overview: The plate was washed twice before adding standards, samples, and blank controls. Fifty microliters of each standard or sample were added to their respective wells, followed immediately by the same amount of a biotin-labeled antibody. The plate was gently tapped for 1 min to ensure thorough mixing and then incubated at 37 °C for 45 min. After this, the plate was washed three times, with each wash lasting 1 min. Subsequently, 100 µL of SABC working solution was added to each well, and the plate was incubated statically at 37 °C for 30 min, then washed five times, each wash lasting 1 min. Next, 90 µL of TMB substrate solution was added, and the plate was incubated at 37 °C for 10–20 min while accurate color development was carefully monitored. The reaction was stopped by adding 50 µL of stop solution. Absorbance was measured at 450 nm using a Smart Microplate Reader (SMR16, USCN KIT, Inc., Wuhan, China). All measurements were conducted in triplicate, and the results were expressed as the mean relative to the untreated control group.

### 2.9. In Vitro Wound Healing Model

HaCaT cells were seeded in six-well plates (10^6^ cells/well) and cultured at 37 °C with 5% CO_2_ for 24 h until ~90% confluence. Cells were then scratched with a 200 μL pipette tip to create a uniform wound and washed with PBS to remove debris. They were then incubated in a serum-free medium, with or without pretreatment with CFS-LS at various concentrations for 24 h. Migration was monitored under an inverted microscope at 50× magnification, and images were taken at 0 h and 24 h post-wounding. Experiments were performed in triplicate, analyzing at least three fields per condition. Wound closure percentage was calculated by measuring the remaining cell-free area relative to the initial wound using ImageJ software version 1.53a (National Institutes of Health, Brussels, Belgium) [26].

### 2.10. Expression Analysis of Collagen Type I and Wound Healing-Related Proteins

HaCaT cells were seeded in six-well plates (5 × 10^5^ cells/well) and incubated at 37 °C with 5% CO_2_ for 24 h; they were then treated with various concentrations of CFS-LS in serum-free medium for an additional 24 h. Conditioned media were collected and centrifuged at 1500× *g* for 20 min. Collagen type I levels in the supernatants (100 μL) were quantified using a human collagen type I ELISA kit (ELK Biotechnology, Sugar Land, TX, USA). The ELISA procedure was performed according to the manufacturer’s instructions. After equilibrating the kit at room temperature, 100 μL of standard working buffer or sample was added to each well and incubated at 37 °C for 80 min. The plate was then washed three times with 200 μL of 1× Wash Buffer per well and blotted dry. Subsequently, 100 μL of biotinylated antibody working solution (1×) was added to each well and incubated at 37 °C for 50 min. After another three washes with 1× Wash Buffer, 100 μL of 1× streptavidin–HRP working solution was added to each well and incubated at 37 °C for 50 min. The plate was then washed five times, blotted dry, and 90 μL of TMB substrate solution was added to each well, followed by incubation at 37 °C for 20 min in the dark. The reaction was stopped by adding 50 μL of stop solution. Absorbance was measured at 450 nm using a Smart Microplate Reader (SMR16, USCN KIT, Inc., China) [27]. Results represent the mean of triplicates relative to untreated controls. For protein analysis, cells were lightly scraped with a pipette tip, and the plates were returned to the incubator for 10 min. The collected cell lysate was subjected to immunoblotting.

### 2.11. Statistical Analysis

Comparisons among multiple groups were performed using one-way ANOVA followed by appropriate post hoc tests to identify significant differences. Differences between the two groups were assessed using Student’s t-test. Results are presented as mean ± standard error of the mean (SEM). A *p*-value < 0.05 was considered significant.

## 3. Results

### 3.1. Effect of CFS-LS on Melanin Production in B16F10 Cells

**Figure 1:**
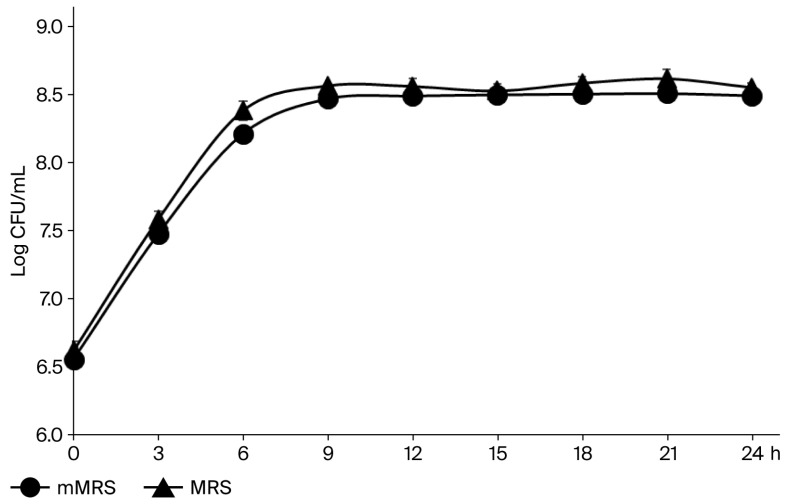
Growth curves of LS cultured in MRS and mMRS broth at 37 °C for 24 h. Data represent mean ± SEM (n = 3).

Although standard MRS broth typically contains Tween 80, which can affect mammalian cells, we assessed LS growth in a modified MRS broth without Tween 80 (mMRS). Since growth in both media was similar (Figure 1), mMRS was used for all subsequent experiments. Cell viability assays revealed that the half-maximal inhibitory concentration (IC_50_) of CFS-LS for B16F10 cells was 32.7% at 24 h and 21.1% at 48 h. For HaCaT cells, the IC_50_ was 33.0% at 24 h. These IC_50_ values are essential for determining the appropriate dosages in our subsequent experiments.

**Figure 2:**
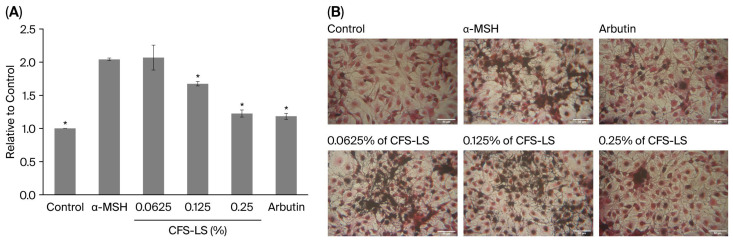
Effect of CFS-LS on melanin production in B16F10 cells, evaluated via melanin content (**A**) and Fontana–Masson staining (**B**). Cells were co-treated with α-MSH (1 μM), CFS-LS, or arbutin (300 μM) for 24 h. After treatment, melanin content was quantified, and stained melanin (black) and nuclei (pink) were visualized under an inverted light microscope (100×, scale bar = 50 μm). Data represent mean ± SEM (n = 3). * *p* < 0.05 vs. α-MSH group.

To assess the anti-melanogenic effect of CFS-LS, α-MSH was used to stimulate melanin production, and arbutin was included as a reference inhibitor. CFS-LS was evaluated at concentrations ranging from 0.0625% to 0.25%, with each successive concentration representing a two-fold dilution. A melanin content assay showed that CFS-LS at 0.125% significantly inhibited melanin synthesis (Figure 2A). At its highest concentration (0.25%), its inhibitory effect was comparable to that of arbutin. These quantitative results were visually confirmed via Fontana–Masson staining, which showed a clear reduction in melanin granules in CFS-LS-treated cells at concentrations of 0.125% or higher (Figure 2B).

### 3.2. Effect of CFS-LS on Melanogenesis-Related Protein Expression in B16F10 Cells

**Figure 3:**
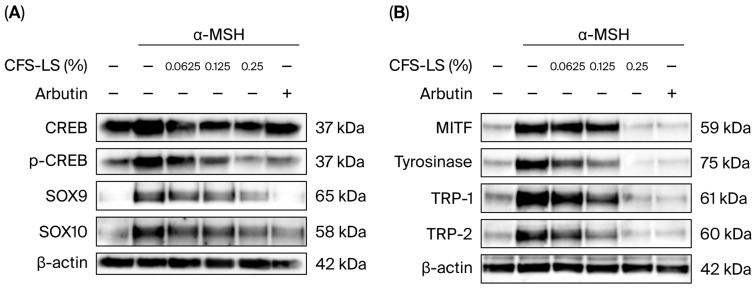
Effect of CFS-LS on melanogenesis-related protein expression in B16F10 cells after 24 h (**A**) and 48 h (**B**) of treatment. Cells were co-treated with 1 μM of α-MSH, indicated concentrations of CFS-LS, or 300 μM of arbutin. Protein expression levels were analyzed via immunoblotting. Immunoblots are representative of three independent experiments.

**Figure 4:**
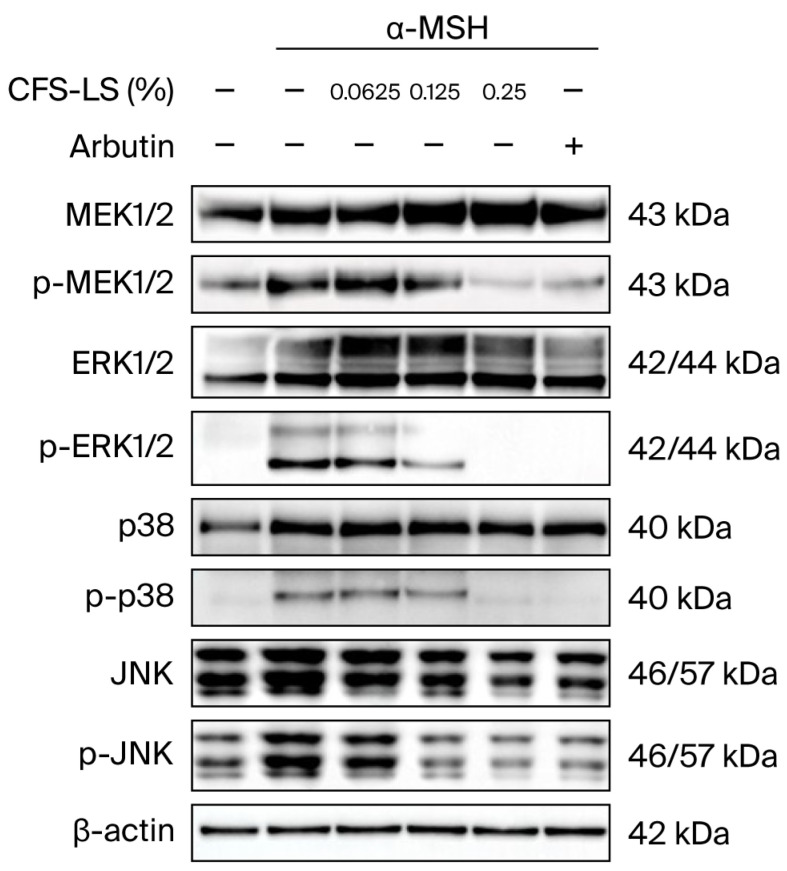
Effect of CFS-LS on MAPK signaling in B16F10 cells. Cells were co-treated with 1 μM of α-MSH, indicated concentrations of CFS-LS, or 300 μM of arbutin for 24 or 48 h. MEK expression was assessed at 24 h, and ERK, JNK, and p38 at 48 h via immunoblotting. Immunoblots are representative of three independent experiments.

To elucidate the molecular mechanisms by which CFS-LS inhibits melanin synthesis, its influence on key proteins was investigated. In B16F10 cells co-treated with α-MSH and 0.25% CFS-LS, the expression of key melanogenic enzymes and transcription factors—including MITF, tyrosinase, tyrosinase-related protein 1 (TRP-1), and tyrosinase-related protein 2 (TRP-2)—was found to be significantly reduced (Figure 3B). Furthermore, we examined the effect of CFS-LS on upstream signaling pathways. It was found that 0.25% CFS-LS inhibited the phosphorylation of CREB (p-CREB) (Figure 3A), a key transcription factor in the PKA/CREB pathway. The expression of other key transcription factors in this pathway, SOX9 and SOX10, was also downregulated (Figure 3B). In addition to the PKA/CREB pathway, we found that 0.25% CFS-LS significantly suppressed the phosphorylation of MEK, ERK, p38, and JNK in the MAPK pathway, indicating that CFS-LS modulates both the PKA/CREB and MAPK signaling cascades to exert its anti-melanogenic effects (Figure 4).

### 3.3. Effect of CFS-LS on UVA-Induced Photodamage in HaCaT Cells

**Figure 5:**
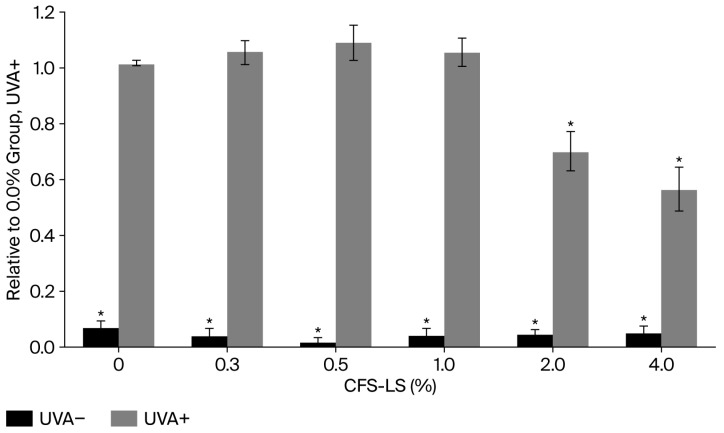
Effect of CFS-LS pretreatment on UVA-induced ROS levels in HaCaT cells. Cells were pretreated with indicated concentrations of CFS-LS for 24 h, followed by UVA exposure (5 J/cm^2^). Intracellular ROS levels were measured after exposure. Data represent mean ± SEM (n = 3). * *p* < 0.05 vs. UVA group without CFS-LS.

Given that UV radiation is a major contributor to skin photodamage, the protective effects of CFS-LS were investigated in HaCaT keratinocytes. The cells were pretreated with various concentrations of CFS-LS for 24 h before being exposed to UVA (5 J/cm^2^) to induce oxidative stress. As shown in Figure 5, CFS-LS demonstrated a dose-dependent protective effect against UVA-induced intracellular ROS generation. Specifically, at concentrations of 2% and 4%, it significantly reduced intracellular ROS levels by 30% and 43%, respectively.

**Figure 6:**
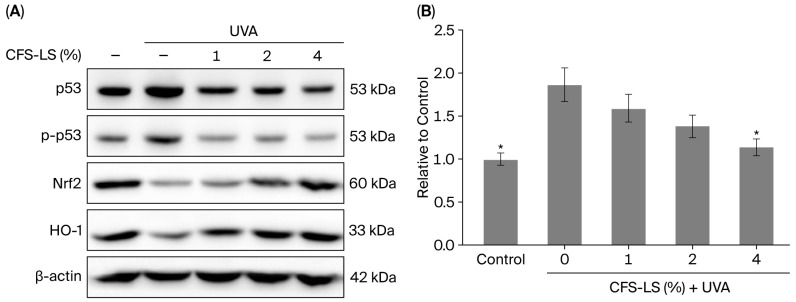
Analysis of protein expression (**A**) and α-MSH levels (**B**) in UVA-irradiated HaCaT cells. The cells were seeded in six-well plates (3 × 10^5^ cells/well) and incubated for 24 h, then pretreated with various concentrations of CFS-LS for an additional 24 h, followed by UVA irradiation at 5 J/cm^2^. After irradiation, they were returned to the incubator for 30 min. Total protein was extracted and analyzed via immunoblotting, while culture supernatants were collected for α-MSH level measurement. The representative blots in (**A**) show protein expression changes. The data in (**B**) are presented as mean ± SEM (n = 3). * *p* < 0.05 vs. UVA group without CFS-LS.

Further analysis of protein expression revealed that CFS-LS modulates multiple signaling pathways in response to UVA. As shown in Figure 6A, CFS-LS at 1% suppressed the expression of phosphorylated p53 (p-p53), a marker of DNA damage. At concentrations of ≥2%, it also restored the UVA-suppressed expression of antioxidant proteins Nrf-2 and heme oxygenase-1 (HO-1). Furthermore, CFS-LS at 4% reduced the levels of α-MSH, a hormone linked to melanogenesis (Figure 6B).

### 3.4. Effect of CFS-LS on Skin Wound Healing in HaCaT Cells

**Figure 7:**
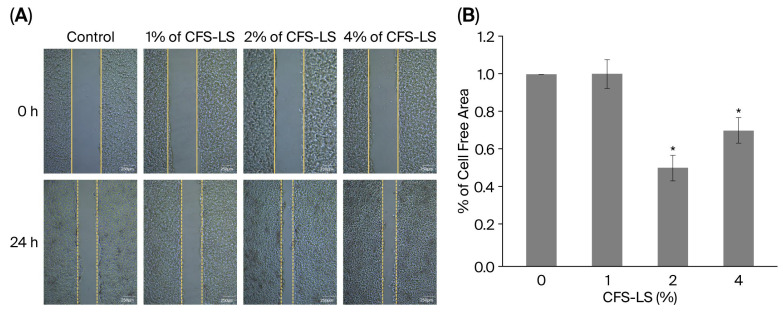
Effect of CFS-LS on wound healing in HaCaT cells. (**A**) Representative images, and (**B**) quantification of the cell-free area. Cells were pretreated with various concentrations of CFS-LS for 24 h, then scratched and incubated for an additional 24 h. Cell migration was assessed with light microscopy (50×, scale bar = 250 μm). Wound closure was quantified by measuring the remaining cell-free area, expressed as a percentage of the initial wound area. Data are presented as mean ± SEM (n = 3). * *p* < 0.05 vs. untreated (0%) group.

Given the critical role of keratinocytes in skin wound repair, the impact of CFS-LS on re-epithelialization processes was investigated in HaCaT cells. As presented in Figure 7A, the treatment with CFS-LS at concentrations of ≥2% significantly promoted HaCaT cell migration in the scratch assay. Quantification of the cell-free area (Figure 7B) revealed a concentration-dependent effect. Specifically, treatment with 2% CFS-LS resulted in a 50% closure of the cell-free area, while 4% increased the coverage to 70%, both of which were significantly greater than the control group.

**Figure 8:**
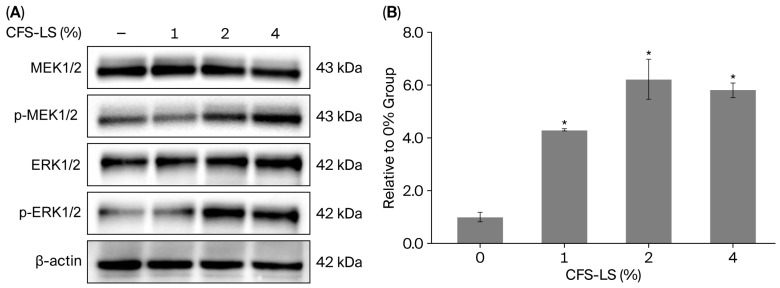
Effect of CFS-LS on MAPK signaling (**A**) and collagen type I synthesis (**B**) in HaCaT cells. For MAPK analysis, cells were pretreated with various concentrations of CFS-LS for 24 h, scratched, and then incubated for 10 min before lysis. Protein levels were assessed via immunoblotting, and representative blots showing changes in protein expression are presented in (**A**). For collagen analysis, cells received the same pretreatment; culture media were collected, and collagen type I levels were measured with ELISA. Data in (**B**) represent mean ± SEM (n = 3). * *p* < 0.05 vs. the untreated (0%) group.

Further investigation into the molecular mechanisms of this pro-migratory effect revealed that CFS-LS modulates the MAPK signaling pathway. As shown in Figure 8A, pretreatment with CFS-LS (at concentrations of ≥2%) increased the phosphorylation of MEK1/2 (pMEK1/2) and ERK1/2 (pERK1/2). Furthermore, CFS-LS treatment also led to a significant elevation in collagen type I levels at concentrations of ≥1% (Figure 8B), a key component for tissue regeneration.

## 4. Discussion

*P. acidophilus*, a common probiotic found in fermented foods and the gut, is known for its wide-ranging health benefits, including improving gut balance, fighting pathogens, boosting immunity, and potentially easing allergies [28,29,30]. This study explored the skin-related advantages of a cell-free supernatant derived from a *P. acidophilus* strain. The findings indicate that CFS-LS effectively reduces melanin production in B16F10 cells, mitigates UVA-induced oxidative stress in HaCaT cells, and aids in wound healing by promoting keratinocyte migration and collagen synthesis. These results suggest that the benefits of *P. acidophilus* LS extend beyond the live bacteria themselves, highlighting the potential of its soluble factors, such as organic acids, bacteriocins, and peptides, in new dermatological applications.

A major challenge in postbiotic research is the chemical complexity of these preparations, which contain inanimate microbial cells, cell fragments, and diverse metabolites [31]. This complexity, along with limitations of techniques like NMR, GC-MS, and LC-MS, hinders comprehensive chemical characterization and precise identification of active compounds [31,32]. Moreover, postbiotic bioactivities often arise from the synergistic effects of multiple components rather than single molecules, making isolation difficult [33]. Therefore, most studies, including ours, focus first on demonstrating bioactivities, while detailed chemical isolation constitutes future work.

The need for safe and effective inhibitors against excessive melanin production is significant. CFS-LS demonstrated strong anti-melanogenic potential by significantly inhibiting α-MSH-induced melanin synthesis, an effect comparable to arbutin. This reduction in melanin content is mechanistically supported by CFS-LS’s ability to downregulate crucial melanogenic enzymes such as tyrosinase, TRP-1, and TRP-2. Furthermore, CFS-LS inhibited the expression of p-CREB and MITF, the master regulator of melanogenesis, along with its cooperative transcription factors SOX9 and SOX10 [3,9,34]. These findings indicate that CFS-LS exerts transcriptional control over melanin production. Notably, CFS-LS also suppressed the phosphorylation of MEK, ERK, p38, and JNK. Given that MITF’s activity is regulated by both the PKA/CREB and MAPK pathways [35,36], these results collectively suggest that CFS-LS attenuates melanogenesis via a dual mechanism, offering a profound, broad inhibitory effect.

UVA radiation is a primary driver of skin aging and hyperpigmentation, causing oxidative stress and DNA damage [6,7,8]. Our study found that CFS-LS markedly reduced UVA-induced intracellular ROS in HaCaT cells, acting as an antioxidant. This decrease in oxidative stress likely prevents the subsequent activation of p53 and production of α-MSH, key mediators in the UVA-induced melanogenesis and damage pathway [9]. Moreover, CFS-LS’s capacity to restore UVA-suppressed Nrf-2 and HO-1 expression provides a clear molecular basis for its antioxidant benefits. Given Nrf-2’s central role in orchestrating cellular antioxidant defenses [37], this suggests that CFS-LS boosts the cell’s inherent ability to combat UV-induced oxidative damage, thus offering significant photodamage protection. A study on the fermented supernatant from a *Cutibacterium acnes* strain (CCSM0331) provides a possible explanation for the types of active components involved in our study. This strain’s supernatant was found to contain short-chain fatty acids (SCFAs) such as fruit acids and propionic acid, along with antioxidant enzymes including glutathione peroxidase (GSH-Px) and total superoxide dismutase (T-SOD). These compounds demonstrate strong antioxidant activity by activating the Nrf-2 pathway to reduce ROS [38], supporting skin health and anti-aging effects. Although our study did not isolate specific marker compounds, the findings from CCSM0331 offer a valuable insight into the potential bioactive ingredients responsible for the observed effects.

Keratinocytes are indispensable for efficient skin wound healing via re-epithelialization, encompassing proliferation, migration, and differentiation. These processes are regulated by MAPK pathways [39] and modulated by signaling involving growth factors, chemokines, and extracellular matrix remodeling, including collagen synthesis and metalloproteases [40]. Our findings show that CFS-LS significantly enhanced HaCaT cell migration in a scratch assay, suggesting its potential to accelerate re-epithelialization. This pro-migratory effect was mechanistically linked to increased pMEK1/2 and pERK1/2, consistent with the ERK pathway’s known role in keratinocyte migration during wound healing [39]. Moreover, CFS-LS significantly elevated collagen type I levels, a crucial component for extracellular matrix deposition, tensile strength, and proper tissue regeneration. Thus, CFS-LS promotes not only cell migration but also key aspects of tissue repair.

This study suggests that CFS-LS, considered a kind of postbiotic, holds significant potential as a multi-functional agent for skin protection and repair. By possibly regulating MAPK signaling in keratinocytes and melanocytes, CFS-LS may mitigate UV-induced oxidative stress, enhance wound healing, and prevent hyperpigmentation. Its putative capacity to support skin barrier integrity further highlights its relevance for dermatological and cosmetic applications.

More specifically, the benefits of CFS-LS are multifaceted. It enhances skin repair by stimulating the migration and proliferation of keratinocytes, as well as boosting collagen production, which collectively accelerate wound healing. Concurrently, it reduces melanin formation in pigment-producing cells, thereby decreasing the risk of post-injury dark spots and uneven pigmentation. The antioxidant properties of CFS-LS also create a healthy environment for healing, reducing oxidative stress and inflammation. These combined effects support not only faster functional recovery but also improved cosmetic appearance, making CFS-LS particularly effective for treating wounds at risk of pigmentation complications.

Furthermore, as a derivative of *P. acidophilus*, a widely studied probiotic, CFS-LS offers natural and safe bioactivity with low irritation potential. Its anti-inflammatory and antimicrobial properties could reduce infection risk, thereby supporting a faster and cleaner healing process. Postbiotics are known to influence immune responses, potentially supporting the recruitment and activity of cells involved in tissue repair while helping to regulate inflammatory cytokines. They may also contribute to maintaining a balanced skin microbiome, which is crucial for a healthy skin barrier [41].

Collectively, these findings indicate that CFS-LS warrants further investigation as a safe and effective candidate for promoting skin health and resilience. It holds great potential for future applications in post-surgical care, dermatological treatments for pigmentation disorders, chronic wound management, anti-aging and brightening skincare, and products aimed at repairing sun-damaged skin.

## 5. Conclusions

The multi-functional properties of CFS-LS observed in this study—encompassing anti-melanogenic, antioxidant, and wound-healing activities—underscore its significant potential as a novel ingredient for dermatological applications. Although individual probiotic strains and their cell-free supernatants have previously exhibited some of these benefits [14,15,16,17,18], the comprehensive effects of CFS-LS across multiple crucial skin health pathways are particularly promising. As a postbiotic composed of bioactive metabolites from probiotic bacteria, CFS-LS offers advantages in safety and formulation stability. This positions it as a versatile candidate for developing advanced cosmetic formulations or therapeutic agents aimed at preventing hyperpigmentation, mitigating photoaging, and accelerating wound recovery. Despite these findings, however, a key challenge remains in connecting these effects to specific chemical components. Therefore, future studies are essential and should employ organic chemistry techniques, including LC-MS/MS, metabolomics, and NMR, to isolate and identify the active compounds. Understanding the precise molecular structures of these compounds is crucial for their optimization and standardization for clinical applications.

## Data Availability

The data used and/or analyzed during the current study are available from the corresponding author upon reasonable request.

## Abbreviations

The following abbreviations are used in this manuscript:

CFS-LS	*Pediococcus acidophilus* strain LS-derived cell-free supernatant
UV	Ultraviolet
ROS	Reactive oxygen species
α-MSH	α-melanocyte-stimulating hormone
MC1R	Melanocortin 1 receptor
MITF	Microphthalmia-associated transcription factor
TRP	Tyrosinase-related proteins
CM-H_2_DCFDA	5-(and-6)-chloromethyl-2′,7′-dichlorodihydrofluorescein diacetate, acetyl ester
NMR	Nuclear magnetic resonance
GC-MS	Gas chromatography–mass spectrometry
LC-MS	Liquid chromatography–mass spectrometry
